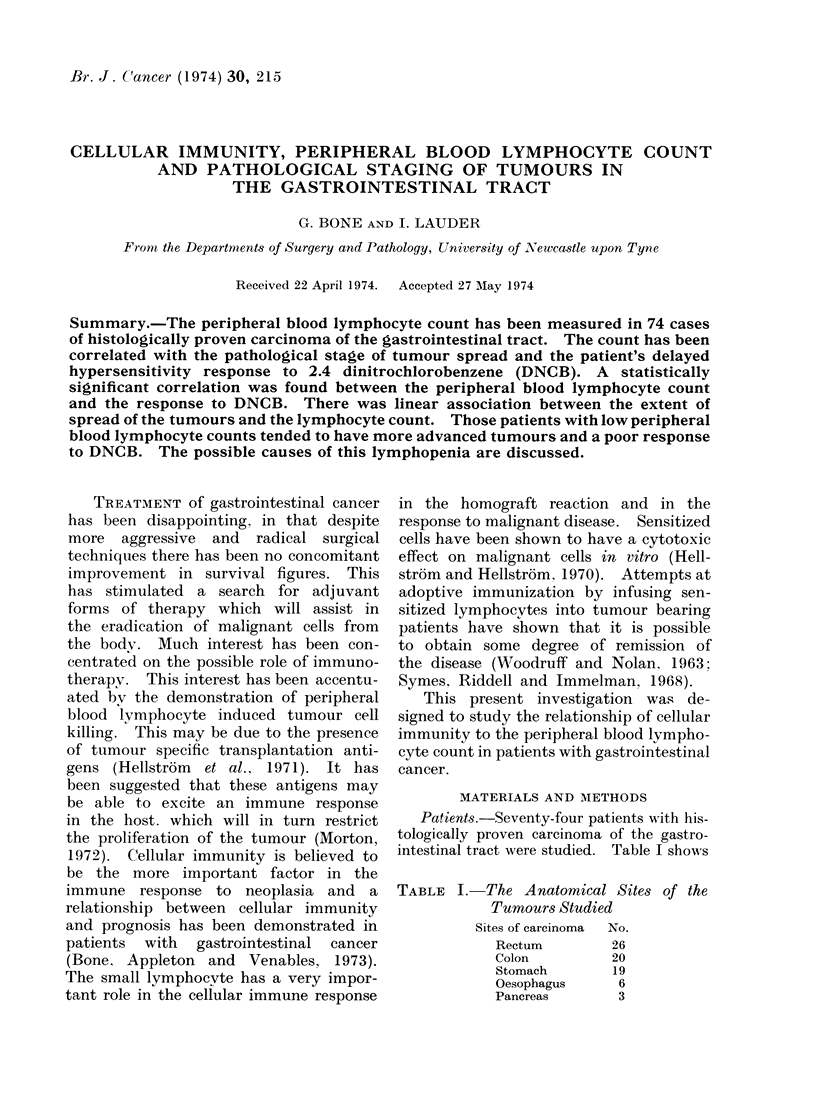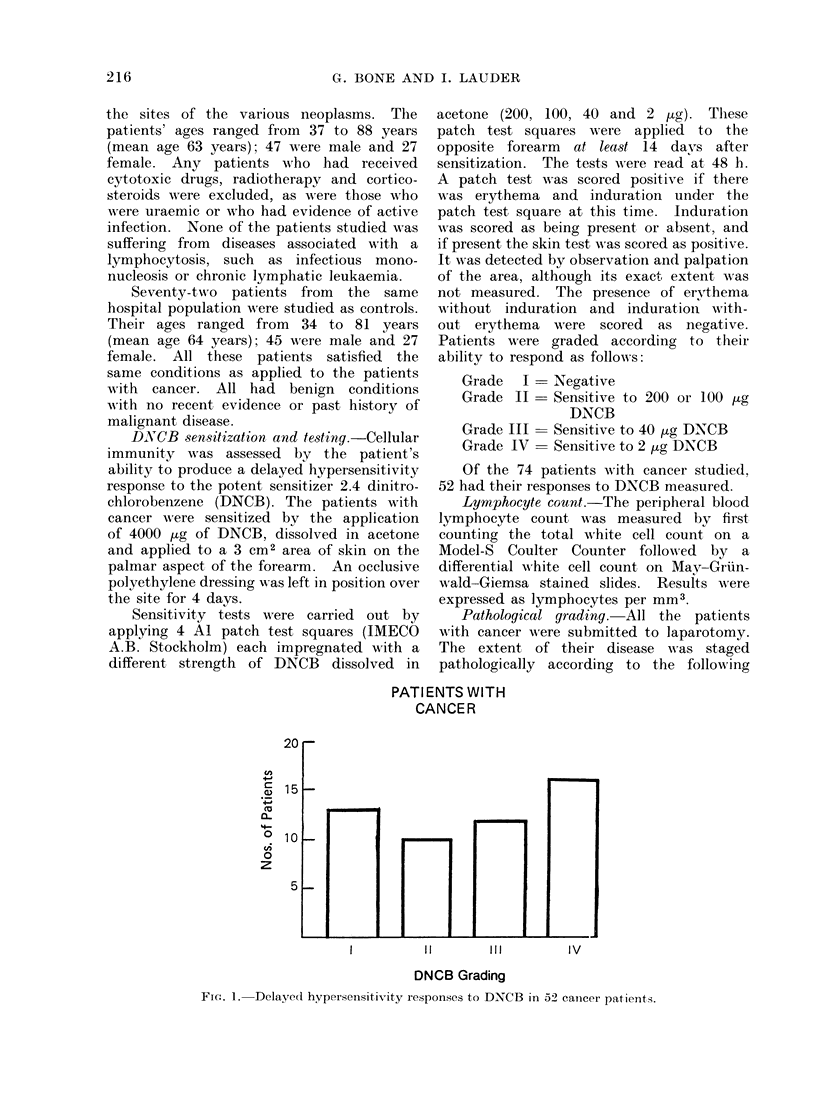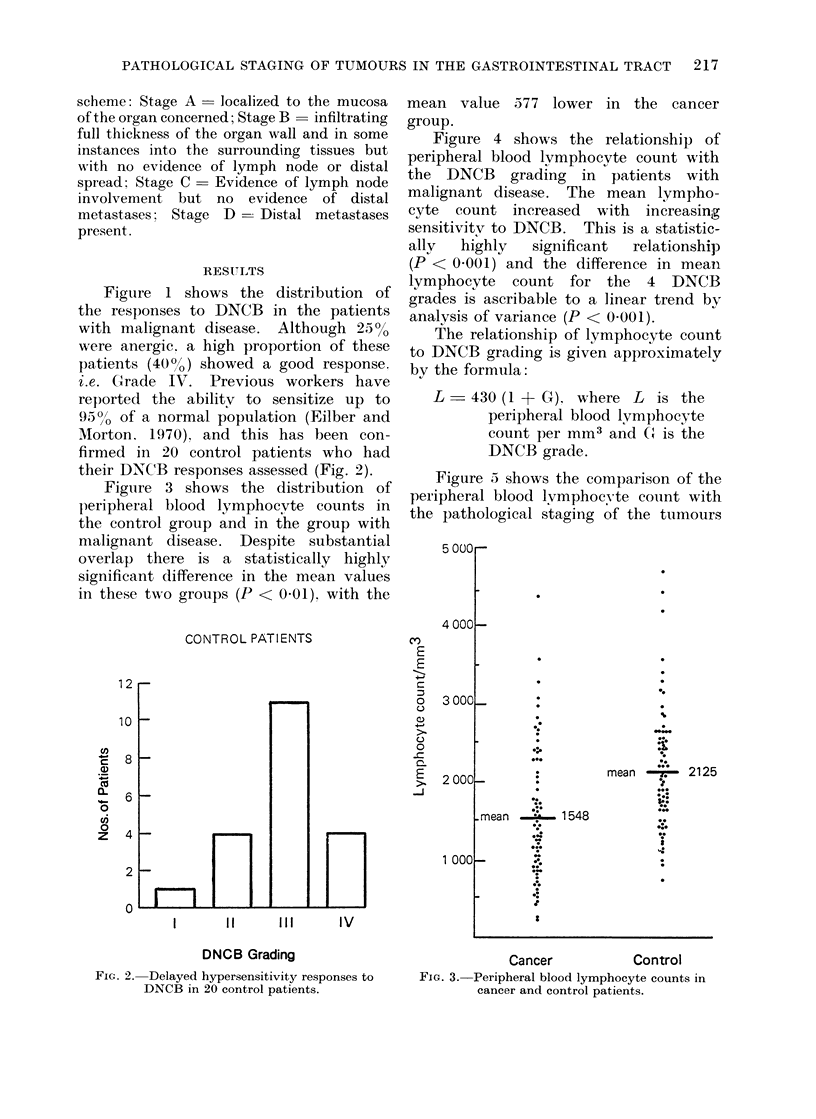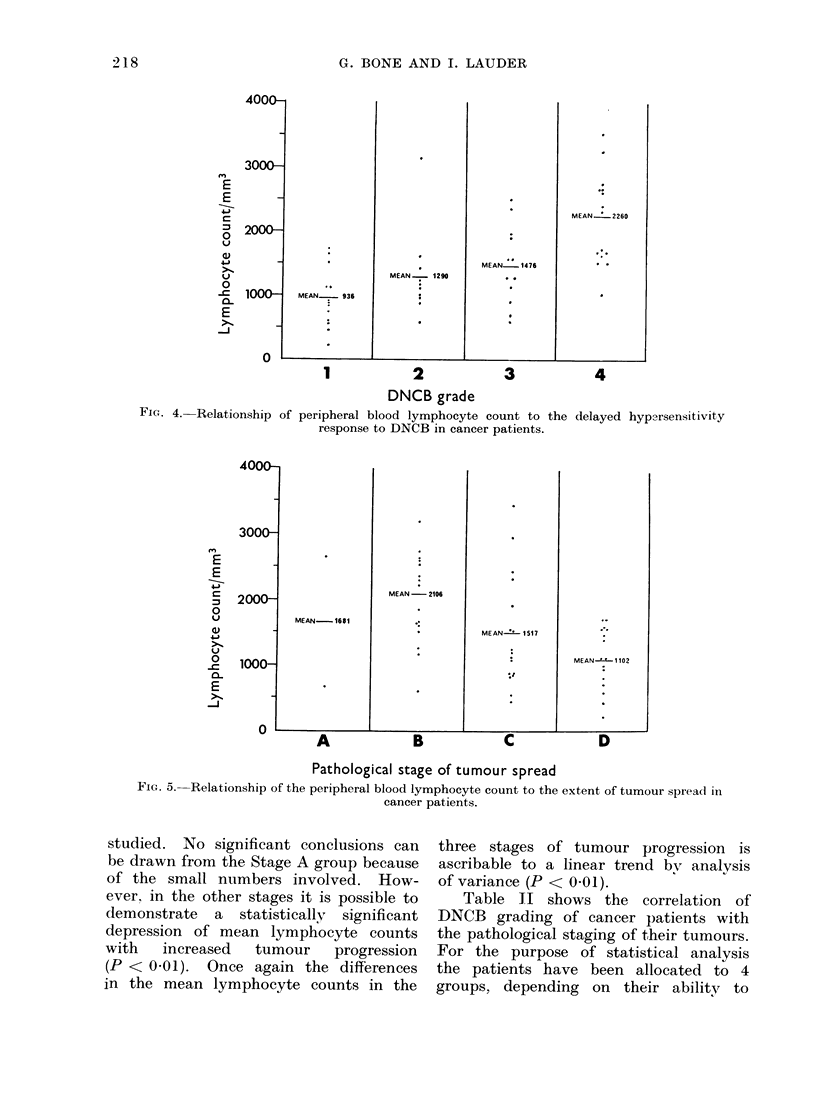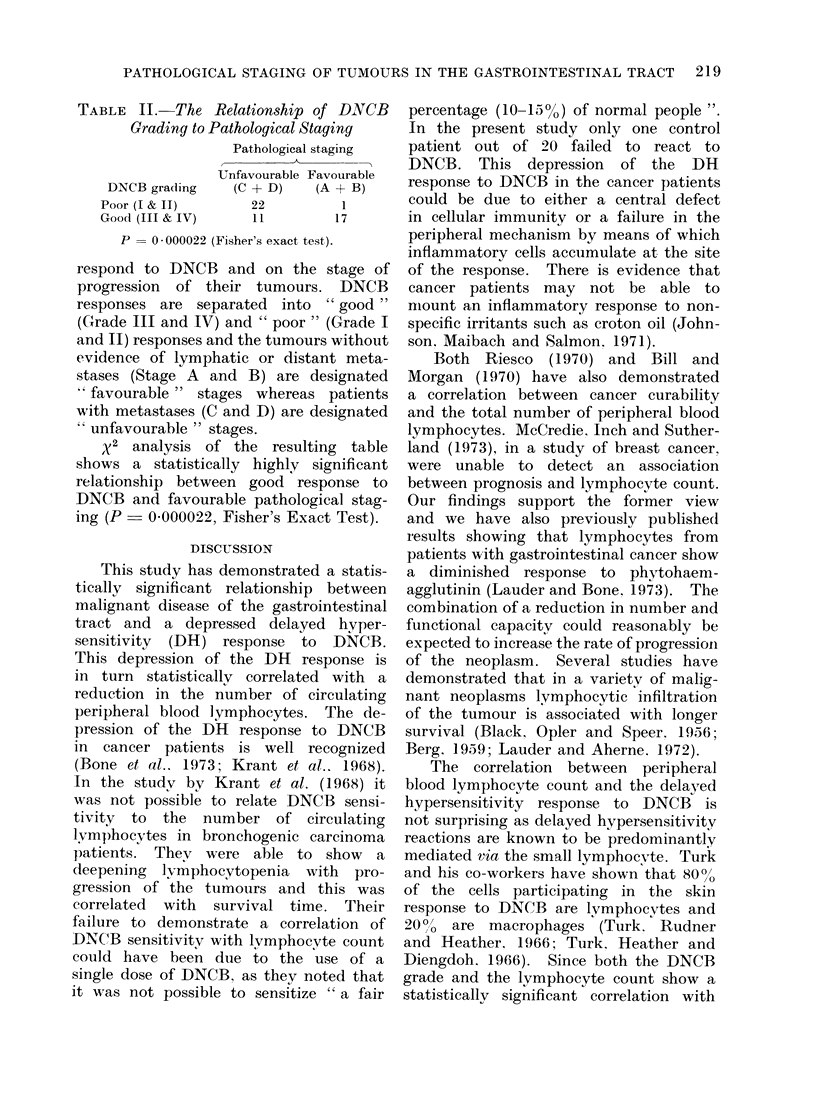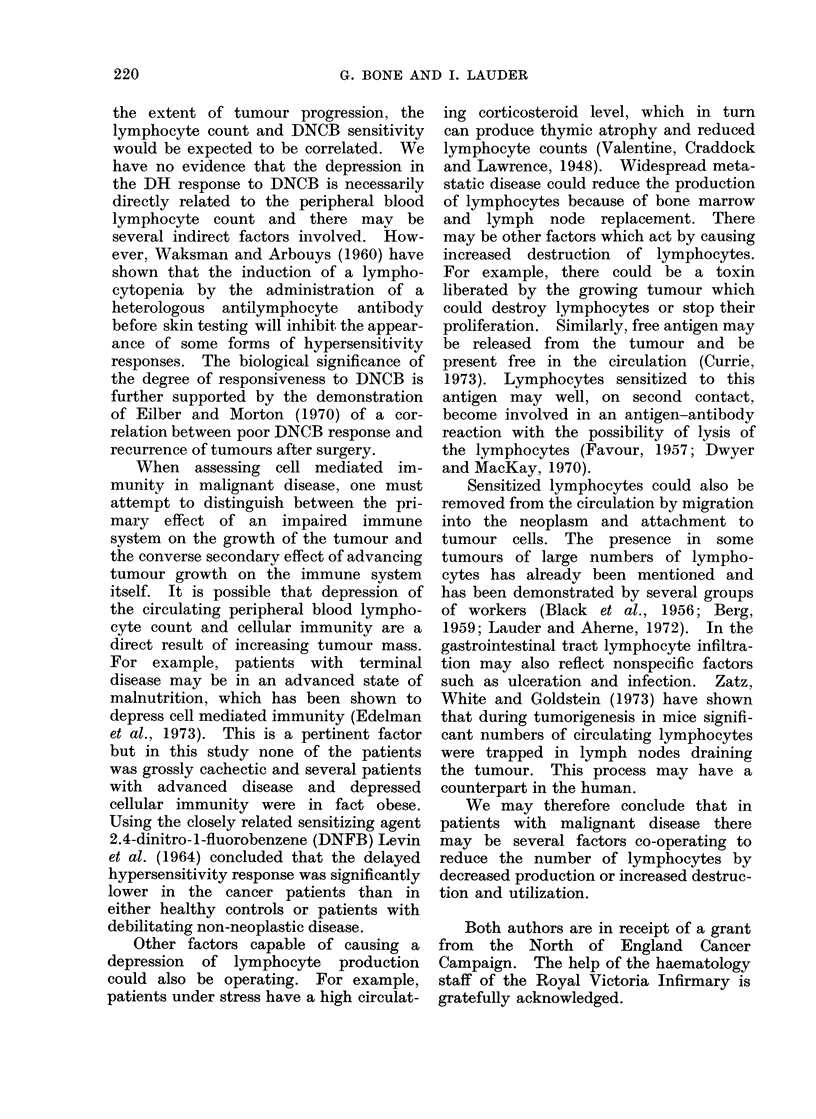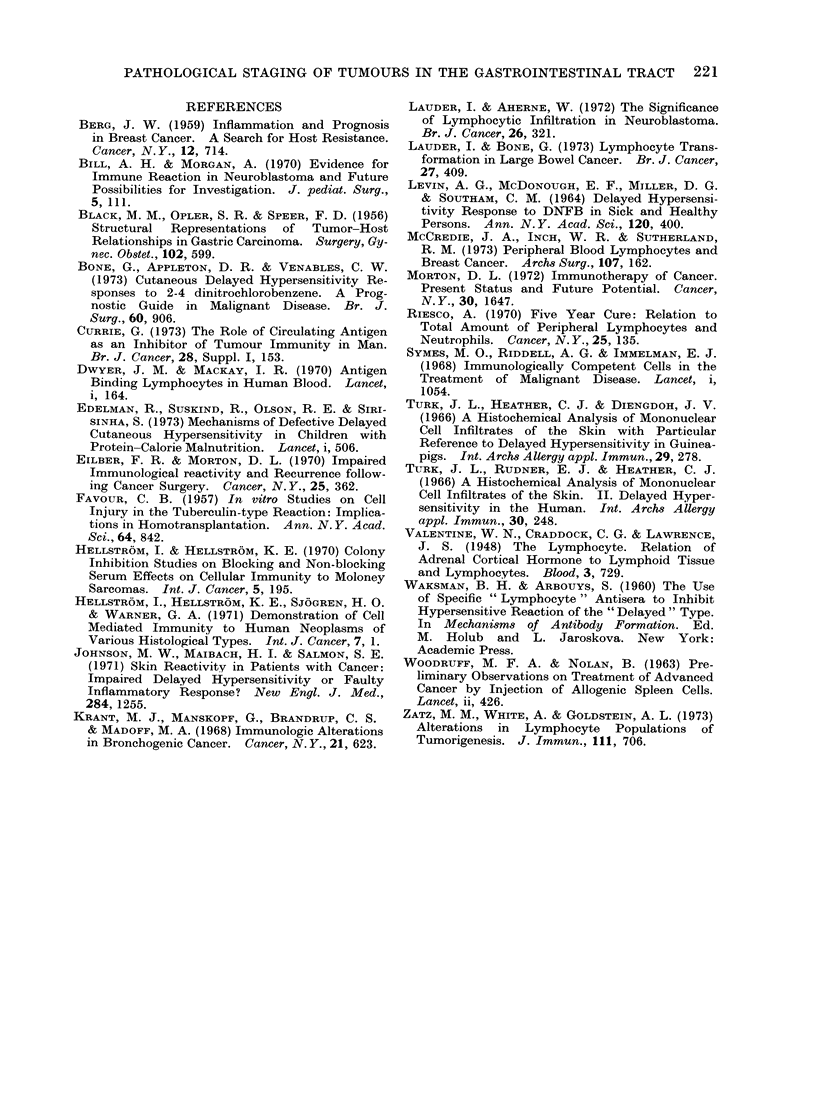# Cellular Immunity, Peripheral Blood Lymphocyte Count and Pathological Staging of Tumours in the Gastrointestinal Tract

**DOI:** 10.1038/bjc.1974.184

**Published:** 1974-09

**Authors:** G. Bone, I. Lauder

## Abstract

The peripheral blood lymphocyte count has been measured in 74 cases of histologically proven carcinoma of the gastrointestinal tract. The count has been correlated with the pathological stage of tumour spread and the patient's delayed hypersensitivity response to 2.4 dinitrochlorobenzene (DNCB). A statistically significant correlation was found between the peripheral blood lymphocyte count and the response to DNCB. There was linear association between the extent of spread of the tumours and the lymphocyte count. Those patients with low peripheral blood lymphocyte counts tended to have more advanced tumours and a poor response to DNCB. The possible causes of this lymphopenia are discussed.


*Br. J. Cancer* (1974) **30**, 215

### CELLULAR IMMUNITY, PERIPHERAL BLOOD LYMPHOCYTE COUNT AND PATHOLOGICAL STAGING OF TUMOURS IN THE GASTROINTESTINAL TRACT

G. BONE AND I. LAUDER

*From the Departments of Surgery and Pathology, University of Newcastle upon Tyne*

Received 22 April 1974. Accepted 27 May 1974

**Summary.—The peripheral blood lymphocyte count has been measured in 74 cases of histologically proven carcinoma of the gastrointestinal tract. The count has been correlated with the pathological stage of tumour spread and the patient's delayed hypersensitivity response to 2.4 dinitrochlorobenzene (DNCB). A statistically significant correlation was found between the peripheral blood lymphocyte count and the response to DNCB. There was linear association between the extent of spread of the tumours and the lymphocyte count. Those patients with low peripheral blood lymphocyte counts tended to have more advanced tumours and a poor response to DNCB. The possible causes of this lymphopenia are discussed.**

TREATMENT of gastrointestinal cancer has been disappointing, in that despite more aggressive and radical surgical techniques there has been no concomitant improvement in survival figures. This has stimulated a search for adjuvant forms of therapy which will assist in the eradication of malignant cells from the body. Much interest has been concentrated on the possible role of immunotherapy. This interest has been accentuated by the demonstration of peripheral blood lymphocyte induced tumour cell killing. This may be due to the presence of tumour specific transplantation antigens (Hellström *et al.*, 1971). It has been suggested that these antigens may be able to excite an immune response in the host, which will in turn restrict the proliferation of the tumour (Morton, 1972). Cellular immunity is believed to be the more important factor in the immune response to neoplasia and a relationship between cellular immunity and prognosis has been demonstrated in patients with gastrointestinal cancer (Bone, Appleton and Venables, 1973). The small lymphocyte has a very important role in the cellular immune response in the homograft reaction and in the response to malignant disease. Sensitized cells have been shown to have a cytotoxic effect on malignant cells *in vitro* (Hellström and Hellström, 1970). Attempts at adoptive immunization by infusing sensitized lymphocytes into tumour bearing patients have shown that it is possible to obtain some degree of remission of the disease (Woodruff and Nolan, 1963; Symes, Riddell and Immelman, 1968).

This present investigation was designed to study the relationship of cellular immunity to the peripheral blood lymphocyte count in patients with gastrointestinal cancer.

### MATERIALS AND METHODS

*Patients.*—Seventy-four patients with histologically proven carcinoma of the gastrointestinal tract were studied. Table I shows

TABLE I.—*The Anatomical Sites of the Tumours Studied*

| Sites of carcinoma | No. |
|---|---|
| Rectum | 26 |
| Colon | 20 |
| Stomach | 19 |
| Oesophagus | 6 |
| Pancreas | 3 |

the sites of the various neoplasms. The patients' ages ranged from 37 to 88 years (mean age 63 years); 47 were male and 27 female. Any patients who had received cytotoxic drugs, radiotherapy and corticosteroids were excluded, as were those who were uraemic or who had evidence of active infection. None of the patients studied was suffering from diseases associated with a lymphocytosis, such as infectious mononucleosis or chronic lymphatic leukaemia.

Seventy-two patients from the same hospital population were studied as controls. Their ages ranged from 34 to 81 years (mean age 64 years); 45 were male and 27 female. All these patients satisfied the same conditions as applied to the patients with cancer. All had benign conditions with no recent evidence or past history of malignant disease.

*DNCB sensitization and testing.*—Cellular immunity was assessed by the patient's ability to produce a delayed hypersensitivity response to the potent sensitizer 2.4 dinitrochlorobenzene (DNCB). The patients with cancer were sensitized by the application of 4000 $\mu g$ of DNCB, dissolved in acetone and applied to a 3 $cm^2$ area of skin on the palmar aspect of the forearm. An occlusive polyethylene dressing was left in position over the site for 4 days.

Sensitivity tests were carried out by applying 4 A1 patch test squares (IMECO A.B. Stockholm) each impregnated with a different strength of DNCB dissolved in acetone (200, 100, 40 and 2 $\mu g$). These patch test squares were applied to the opposite forearm *at least* 14 days after sensitization. The tests were read at 48 h. A patch test was scored positive if there was erythema and induration under the patch test square at this time. Induration was scored as being present or absent, and if present the skin test was scored as positive. It was detected by observation and palpation of the area, although its exact extent was not measured. The presence of erythema without induration and induration without erythema were scored as negative. Patients were graded according to their ability to respond as follows:

Grade I = Negative

Grade II = Sensitive to 200 or 100 $\mu g$ DNCB

Grade III = Sensitive to 40 $\mu g$ DNCB

Grade IV = Sensitive to 2 $\mu g$ DNCB

Of the 74 patients with cancer studied, 52 had their responses to DNCB measured.

*Lymphocyte count.*—The peripheral blood lymphocyte count was measured by first counting the total white cell count on a Model-S Coulter Counter followed by a differential white cell count on May–Grünwald–Giemsa stained slides. Results were expressed as lymphocytes per $mm^3$.

*Pathological grading.*—All the patients with cancer were submitted to laparotomy. The extent of their disease was staged pathologically according to the following

FIG. 1.—Delayed hypersensitivity responses to DNCB in 52 cancer patients.

scheme: Stage A = localized to the mucosa of the organ concerned; Stage B = infiltrating full thickness of the organ wall and in some instances into the surrounding tissues but with no evidence of lymph node or distal spread; Stage C = Evidence of lymph node involvement but no evidence of distal metastases; Stage D = Distal metastases present.

### RESULTS

Figure 1 shows the distribution of the responses to DNCB in the patients with malignant disease. Although 25% were anergic, a high proportion of these patients (40%) showed a good response, *i.e.* Grade IV. Previous workers have reported the ability to sensitize up to 95% of a normal population (Eilber and Morton, 1970), and this has been confirmed in 20 control patients who had their DNCB responses assessed (Fig. 2).

Figure 3 shows the distribution of peripheral blood lymphocyte counts in the control group and in the group with malignant disease. Despite substantial overlap there is a statistically highly significant difference in the mean values in these two groups ($P < 0{\cdot}01$), with the mean value 577 lower in the cancer group.

Figure 4 shows the relationship of peripheral blood lymphocyte count with the DNCB grading in patients with malignant disease. The mean lymphocyte count increased with increasing sensitivity to DNCB. This is a statistically highly significant relationship ($P < 0{\cdot}001$) and the difference in mean lymphocyte count for the 4 DNCB grades is ascribable to a linear trend by analysis of variance ($P < 0{\cdot}001$).

The relationship of lymphocyte count to DNCB grading is given approximately by the formula:

$L = 430\,(1 + G)$, where $L$ is the peripheral blood lymphocyte count per mm$^3$ and G is the DNCB grade.

Figure 5 shows the comparison of the peripheral blood lymphocyte count with the pathological staging of the tumours

FIG. 2.—Delayed hypersensitivity responses to DNCB in 20 control patients.

FIG. 3.—Peripheral blood lymphocyte counts in cancer and control patients.

FIG. 4.—Relationship of peripheral blood lymphocyte count to the delayed hypersensitivity response to DNCB in cancer patients.

FIG. 5.—Relationship of the peripheral blood lymphocyte count to the extent of tumour spread in cancer patients.

studied. No significant conclusions can be drawn from the Stage A group because of the small numbers involved. However, in the other stages it is possible to demonstrate a statistically significant depression of mean lymphocyte counts with increased tumour progression ($P < 0{\cdot}01$). Once again the differences in the mean lymphocyte counts in the three stages of tumour progression is ascribable to a linear trend by analysis of variance ($P < 0{\cdot}01$).

Table II shows the correlation of DNCB grading of cancer patients with the pathological staging of their tumours. For the purpose of statistical analysis the patients have been allocated to 4 groups, depending on their ability to

TABLE II.—*The Relationship of DNCB Grading to Pathological Staging*

| DNCB grading | Pathological staging: Unfavourable (C + D) | Favourable (A + B) |
|---|---|---|
| Poor (I & II) | 22 | 1 |
| Good (III & IV) | 11 | 17 |

$P = 0 \cdot 000022$ (Fisher's exact test).

respond to DNCB and on the stage of progression of their tumours. DNCB responses are separated into " good " (Grade III and IV) and " poor " (Grade I and II) responses and the tumours without evidence of lymphatic or distant metastases (Stage A and B) are designated " favourable " stages whereas patients with metastases (C and D) are designated " unfavourable " stages.

$\chi^2$ analysis of the resulting table shows a statistically highly significant relationship between good response to DNCB and favourable pathological staging ($P = 0{\cdot}000022$, Fisher's Exact Test).

### DISCUSSION

This study has demonstrated a statistically significant relationship between malignant disease of the gastrointestinal tract and a depressed delayed hypersensitivity (DH) response to DNCB. This depression of the DH response is in turn statistically correlated with a reduction in the number of circulating peripheral blood lymphocytes. The depression of the DH response to DNCB in cancer patients is well recognized (Bone *et al.*, 1973; Krant *et al.*, 1968). In the study by Krant *et al.* (1968) it was not possible to relate DNCB sensitivity to the number of circulating lymphocytes in bronchogenic carcinoma patients. They were able to show a deepening lymphocytopenia with progression of the tumours and this was correlated with survival time. Their failure to demonstrate a correlation of DNCB sensitivity with lymphocyte count could have been due to the use of a single dose of DNCB, as they noted that it was not possible to sensitize " a fair percentage (10–15%) of normal people ". In the present study only one control patient out of 20 failed to react to DNCB. This depression of the DH response to DNCB in the cancer patients could be due to either a central defect in cellular immunity or a failure in the peripheral mechanism by means of which inflammatory cells accumulate at the site of the response. There is evidence that cancer patients may not be able to mount an inflammatory response to nonspecific irritants such as croton oil (Johnson, Maibach and Salmon, 1971).

Both Riesco (1970) and Bill and Morgan (1970) have also demonstrated a correlation between cancer curability and the total number of peripheral blood lymphocytes. McCredie, Inch and Sutherland (1973), in a study of breast cancer, were unable to detect an association between prognosis and lymphocyte count. Our findings support the former view and we have also previously published results showing that lymphocytes from patients with gastrointestinal cancer show a diminished response to phytohaemagglutinin (Lauder and Bone, 1973). The combination of a reduction in number and functional capacity could reasonably be expected to increase the rate of progression of the neoplasm. Several studies have demonstrated that in a variety of malignant neoplasms lymphocytic infiltration of the tumour is associated with longer survival (Black, Opler and Speer, 1956; Berg, 1959; Lauder and Aherne, 1972).

The correlation between peripheral blood lymphocyte count and the delayed hypersensitivity response to DNCB is not surprising as delayed hypersensitivity reactions are known to be predominantly mediated *via* the small lymphocyte. Turk and his co-workers have shown that 80% of the cells participating in the skin response to DNCB are lymphocytes and 20% are macrophages (Turk, Rudner and Heather, 1966; Turk, Heather and Diengdoh, 1966). Since both the DNCB grade and the lymphocyte count show a statistically significant correlation with the extent of tumour progression, the lymphocyte count and DNCB sensitivity would be expected to be correlated. We have no evidence that the depression in the DH response to DNCB is necessarily directly related to the peripheral blood lymphocyte count and there may be several indirect factors involved. However, Waksman and Arbouys (1960) have shown that the induction of a lymphocytopenia by the administration of a heterologous antilymphocyte antibody before skin testing will inhibit the appearance of some forms of hypersensitivity responses. The biological significance of the degree of responsiveness to DNCB is further supported by the demonstration of Eilber and Morton (1970) of a correlation between poor DNCB response and recurrence of tumours after surgery.

When assessing cell mediated immunity in malignant disease, one must attempt to distinguish between the primary effect of an impaired immune system on the growth of the tumour and the converse secondary effect of advancing tumour growth on the immune system itself. It is possible that depression of the circulating peripheral blood lymphocyte count and cellular immunity are a direct result of increasing tumour mass. For example, patients with terminal disease may be in an advanced state of malnutrition, which has been shown to depress cell mediated immunity (Edelman *et al.*, 1973). This is a pertinent factor but in this study none of the patients was grossly cachectic and several patients with advanced disease and depressed cellular immunity were in fact obese. Using the closely related sensitizing agent 2.4-dinitro-1-fluorobenzene (DNFB) Levin *et al.* (1964) concluded that the delayed hypersensitivity response was significantly lower in the cancer patients than in either healthy controls or patients with debilitating non-neoplastic disease.

Other factors capable of causing a depression of lymphocyte production could also be operating. For example, patients under stress have a high circulating corticosteroid level, which in turn can produce thymic atrophy and reduced lymphocyte counts (Valentine, Craddock and Lawrence, 1948). Widespread metastatic disease could reduce the production of lymphocytes because of bone marrow and lymph node replacement. There may be other factors which act by causing increased destruction of lymphocytes. For example, there could be a toxin liberated by the growing tumour which could destroy lymphocytes or stop their proliferation. Similarly, free antigen may be released from the tumour and be present free in the circulation (Currie, 1973). Lymphocytes sensitized to this antigen may well, on second contact, become involved in an antigen–antibody reaction with the possibility of lysis of the lymphocytes (Favour, 1957; Dwyer and MacKay, 1970).

Sensitized lymphocytes could also be removed from the circulation by migration into the neoplasm and attachment to tumour cells. The presence in some tumours of large numbers of lymphocytes has already been mentioned and has been demonstrated by several groups of workers (Black *et al.*, 1956; Berg, 1959; Lauder and Aherne, 1972). In the gastrointestinal tract lymphocyte infiltration may also reflect nonspecific factors such as ulceration and infection. Zatz, White and Goldstein (1973) have shown that during tumorigenesis in mice significant numbers of circulating lymphocytes were trapped in lymph nodes draining the tumour. This process may have a counterpart in the human.

We may therefore conclude that in patients with malignant disease there may be several factors co-operating to reduce the number of lymphocytes by decreased production or increased destruction and utilization.

Both authors are in receipt of a grant from the North of England Cancer Campaign. The help of the haematology staff of the Royal Victoria Infirmary is gratefully acknowledged.

### REFERENCES

Berg, J. W. (1959) Inflammation and Prognosis in Breast Cancer. A Search for Host Resistance. *Cancer, N.Y.*, **12**, 714.

Bill, A. H. & Morgan, A. (1970) Evidence for Immune Reaction in Neuroblastoma and Future Possibilities for Investigation. *J. pediat. Surg.*, **5**, 111.

Black, M. M., Opler, S. R. & Speer, F. D. (1956) Structural Representations of Tumor–Host Relationships in Gastric Carcinoma. *Surgery, Gynec. Obstet.*, **102**, 599.

Bone, G., Appleton, D. R. & Venables, C. W. (1973) Cutaneous Delayed Hypersensitivity Responses to 2-4 dinitrochlorobenzene. A Prognostic Guide in Malignant Disease. *Br. J. Surg.*, **60**, 906.

Currie, G. (1973) The Role of Circulating Antigen as an Inhibitor of Tumour Immunity in Man. *Br. J. Cancer*, **28**, Suppl. I, 153.

Dwyer, J. M. & Mackay, I. R. (1970) Antigen Binding Lymphocytes in Human Blood. *Lancet*, i, 164.

Edelman, R., Suskind, R., Olson, R. E. & Sirisinha, S. (1973) Mechanisms of Defective Delayed Cutaneous Hypersensitivity in Children with Protein–Calorie Malnutrition. *Lancet*, i, 506.

Eilber, F. R. & Morton, D. L. (1970) Impaired Immunological reactivity and Recurrence following Cancer Surgery. *Cancer, N.Y.*, **25**, 362.

Favour, C. B. (1957) *In vitro* Studies on Cell Injury in the Tuberculin-type Reaction: Implications in Homotransplantation. *Ann. N.Y. Acad. Sci.*, **64**, 842.

Hellström, I. & Hellström, K. E. (1970) Colony Inhibition Studies on Blocking and Non-blocking Serum Effects on Cellular Immunity to Moloney Sarcomas. *Int. J. Cancer*, **5**, 195.

Hellström, I., Hellström, K. E., Sjögren, H. O. & Warner, G. A. (1971) Demonstration of Cell Mediated Immunity to Human Neoplasms of Various Histological Types. *Int. J. Cancer*, **7**, 1.

Johnson, M. W., Maibach, H. I. & Salmon, S. E. (1971) Skin Reactivity in Patients with Cancer: Impaired Delayed Hypersensitivity or Faulty Inflammatory Response? *New Engl. J. Med.*, **284**, 1255.

Krant, M. J., Manskopf, G., Brandrup, C. S. & Madoff, M. A. (1968) Immunologic Alterations in Bronchogenic Cancer. *Cancer, N.Y.*, **21**, 623.

Lauder, I. & Aherne, W. (1972) The Significance of Lymphocytic Infiltration in Neuroblastoma. *Br. J. Cancer*, **26**, 321.

Lauder, I. & Bone, G. (1973) Lymphocyte Transformation in Large Bowel Cancer. *Br. J. Cancer*, **27**, 409.

Levin, A. G., McDonough, E. F., Miller, D. G. & Southam, C. M. (1964) Delayed Hypersensitivity Response to DNFB in Sick and Healthy Persons. *Ann. N.Y. Acad. Sci.*, **120**, 400.

McCredie, J. A., Inch, W. R. & Sutherland, R. M. (1973) Peripheral Blood Lymphocytes and Breast Cancer. *Archs Surg.*, **107**, 162.

Morton, D. L. (1972) Immunotherapy of Cancer. Present Status and Future Potential. *Cancer, N.Y.*, **30**, 1647.

Riesco, A. (1970) Five Year Cure: Relation to Total Amount of Peripheral Lymphocytes and Neutrophils. *Cancer, N.Y.*, **25**, 135.

Symes, M. O., Riddell, A. G. & Immelman, E. J. (1968) Immunologically Competent Cells in the Treatment of Malignant Disease. *Lancet*, i, 1054.

Turk, J. L., Heather, C. J. & Diengdoh, J. V. (1966) A Histochemical Analysis of Mononuclear Cell Infiltrates of the Skin with Particular Reference to Delayed Hypersensitivity in Guinea-pigs. *Int. Archs Allergy appl. Immun.*, **29**, 278.

Turk, J. L., Rudner, E. J. & Heather, C. J. (1966) A Histochemical Analysis of Mononuclear Cell Infiltrates of the Skin. II. Delayed Hypersensitivity in the Human. *Int. Archs Allergy appl. Immun.*, **30**, 248.

Valentine, W. N., Craddock, C. G. & Lawrence, J. S. (1948) The Lymphocyte. Relation of Adrenal Cortical Hormone to Lymphoid Tissue and Lymphocytes. *Blood*, **3**, 729.

Waksman, B. H. & Arbouys, S. (1960) The Use of Specific "Lymphocyte" Antisera to Inhibit Hypersensitive Reaction of the "Delayed" Type. In *Mechanisms of Antibody Formation.* Ed. M. Holub and L. Jaroskova. New York: Academic Press.

Woodruff, M. F. A. & Nolan, B. (1963) Preliminary Observations on Treatment of Advanced Cancer by Injection of Allogenic Spleen Cells. *Lancet*, ii, 426.

Zatz, M. M., White, A. & Goldstein, A. L. (1973) Alterations in Lymphocyte Populations of Tumorigenesis. *J. Immun.*, **111**, 706.